# Bacterial consortium construction for inhibition of *Salmonella* in chickens

**DOI:** 10.3389/fmicb.2026.1895137

**Published:** 2026-09-15

**Authors:** Dylan Harris, Torey Looft, Melha Mellata

**Affiliations:** 1 Interdepartmental Microbiology Graduate Program, Iowa State University, Ames, IA, United States; 2 Department of Agriculture Food Safety and Enteric Pathogens, Ames, IA, United States; 3 Food Science and Human Nutrition, Iowa State University, Ames, IA, United States

**Keywords:** bacterial consortia, colonization inhibition, competitive exclusion, defined communities, food safety, poultry (chicken), probiotics, *Salmonella*

## Abstract

*Salmonella enterica* is one of the leading causes of bacterial enteritis in humans with consumption of contaminated poultry products being the leading cause of *Salmonella* infection. *Salmonella* is often considered a commensal organism within the poultry gut due to its asymptomatic carriage in adult birds making it difficult to clear by the host immune system once established. Understanding the niche *Salmonella* occupies will aid in the determination or selection for probiotic organisms with overlapping niche occupation metabolically or spatially for inhibition or exclusion of *Salmonella* within the poultry gut. Probiotics are an often-utilized treatment for reducing the presence of *Salmonella* within the poultry gut or as a preventative measure via colonization inhibition through the properties of competitive exclusion. Competitive exclusion is a well-known and effective strategy against *Salmonella* colonization, especially through the administration of undefined chicken cecal contents, effectively blocking *Salmonella* from establishing within the poultry gut. However, creation of a defined consortium of bacteria capable of replicating an undefined community’s ability to eliminate or prevent the colonization of *Salmonella* has yet to be discovered. Advances in computation and sequencing technologies now allow for rapid metabolic comparisons to find organisms with the greatest overlap with metabolic niche of *Salmonella* paving the way for *in silico* predictions of defined anti-*Salmonella* microbial consortia. This review discusses the niche of *Salmonella* within the poultry gut and strategies for creating, testing, and evaluating microbial consortia for the ability to inhibit pathogens.

## Introduction

*Salmonella* is a Gram-negative genus from the *Enterobacteriaceae* family. This facultative anaerobic and facultative intracellular bacterium is often classified either as typhoidal, the causative agent of enteric fever or typhoid, or non-typhoidal, causing mild to severe gastroenteritis (Lamichhane et al., 2024). Non-typhoidal *Salmonella* will be the focus of this review as it is one of the leading causes of bacterial foodborne illness in humans, often sharing the top position with *Campylobacter,* with both diseases causing gastroenteritis predominantly (Scallan Walter et al., 2019). Due to its high prevalence in the United States, non-typhoidal *Salmonella* is estimated to cost 17.1 billion dollars annually in human treatment costs, loss of quality of life, and premature death in 2023 (Hoffmann et al., 2025). The Centers for Disease Control and Prevention estimates a total 1.02 million infections occur in the United States (U. S.) per year, making *Salmonella* a leading cause of foodborne illness, hospitalization, and death in the U. S. The largest source of *Salmonella* infections in humans is the consumption of contaminated poultry products, estimated to comprise 18.9% of infections (Interagency Food Safety Analystics Collaboration, 2023). *Salmonella* is a frequent pathogen found in chicken products, contaminating approximately 1 in 25 packages of chicken in the U. S. making its control a particularly pressing (U.S. Food and Drug Administration, 2024).

*Salmonella* is often considered a commensal organism within poultry, though in early life, it can exhibit subclinical pathogenic traits such as invasion of the intestinal epithelium and induction of inflammatory responses (Ijaz et al., 2021; Gart et al., 2016). *Salmonella* typically colonizes young birds when their immune systems are not fully developed, and before their immature microbiome has developed colonization resistance (Shaji et al., 2023; Foley et al., 2011; Bailey, 1988). Transmission of *Salmonella* within poultry occurs most often via horizontal means through ingestion of infected feces found in dirty bedding, eggshell surface, food, or water, though vertical transmission is known to occur during egg development prior to laying (Byrd et al., 1998; De Reu et al., 2006; Padron, 1990). Infected birds will remain carriers for life, especially when infected at a young age, and shed *Salmonella* within their flock perpetuating the pathogen (Gast et al., 2017). As the use of antibiotics for growth promotion and to inhibit pathogens among livestock and poultry declines, alternative methods for replicating the effects of antibiotics, such as the use of probiotic organisms, have begun to gain popularity, opening up space for the creation of novel bacterial consortiums for inhibition of pathogens (Zhang Y. et al., 2025).

## Subclinical *Salmonella* colonization in poultry

*Salmonella* invades the epithelial lining of the chicken intestinal tract, primarily colonizing the ceca of young chicks. This invasion can trigger a 3-4-day period of inflammation with similar symptomology to a gastrointestinal infection, characterized by an influx of granulocytes to the epithelium. Immune cells are drawn to the site due to the release of pro-inflammatory cytokines and chemokines. As a result, the epithelial tissue shifts to an anabolic metabolism, which depletes local oxygen levels in the epithelium (Withanage et al., 2005; Matulova et al., 2013; Withanage et al., 2004). This change in oxygen levels creates an anaerobic environment that activates the transcription factor hypoxia-inducible factor-α (HIF1α). When HIF1α is activated, it inhibits the mammalian target of rapamycin (mTOR) pathway. This inhibition results in the formation of a localized anti-inflammatory microenvironment by stimulating T regulatory (T_reg_) cells (Clambey et al., 2012; Wouters and Koritzinsky, 2008). In infected chicks, the anti-inflammatory response is enhanced by an upregulation of the Th2 response and an increase expression in anti-inflammatory cytokines such as IL-10 and TGF-β. As a result of these responses, the affected birds continue to shed *Salmonella* for many weeks after the initial infection. This occurs because the host shifts from disease resistance to disease tolerance, which leads to a reduction in inflammation typically seen in the early stages of infection (Withanage et al., 2005; Chaussé et al., 2014; Setta et al., 2012). *Salmonella* can exploit both the canonical and non-canonical TGF-β signaling pathways, as well as non-canonical Wnt signaling pathways. These pathways promote the function of Treg cells while suppressing the function of non-Treg cells reducing the inflammatory response against *Salmonella* (Kogut et al., 2012). Additionally, interferon gamma, which plays a crucial role in eliminating *Salmonella* infections, is down-regulated due to disruptions in the JAK–STAT pathway (Setta et al., 2012; Sadeyen et al., 2004). This immune system suppression allows *Salmonella* to be treated as a commensal organism in the gut, leading to its asymptomatic presence. This shift to being treated as a commensal by the host immune system makes traditional pathogen-focused interventions, such as vaccination, insufficient to completely eliminate the risk of *Salmonella* contamination preharvest. This situation poses a significant risk to human health, especially since broiler chickens are typically slaughtered at approximately 5 weeks of age, which is often before *Salmonella* shedding lessens or ceases. Furthermore, some birds, particularly laying hens, may experience persistent Salmonella infection, jeopardizing the safety of the eggs they lay (Gast et al., 2021; Pande et al., 2016).

## Metabolic and physical niche of *Salmonella*

*Salmonella enterica* is a generalist in terms of metabolism, sharing a core metabolic process with its close relative, *Escherichia coli* (Bock and Sawers, 1996). This shared metabolism allows *Salmonella* to utilize a wide variety of substrates. However, it has adapted its metabolism compared to *E. coli* to promote a more virulent lifestyle in chickens. These adaptations include the ability to use thiosulfate, which is present in an inflamed gut, as an alternative electron acceptor, a capability that *E. coli* does not possess (Meysman et al., 2013). Additionally, *Salmonella* prefers specific carbohydrates for gut colonization, such as galactitol, and it competes for these carbohydrates by challenging other bacteria in the host’s microbiome (Nolle et al., 2017; Eberl et al., 2021). Research has shown that this strategy of increased overlap in carbohydrate utilization between *Salmonella* and the resident microbiome leads to a reduction in *Salmonella* population in the gut (Spragge et al., 2023). Therefore, creating a community of bacteria competing for the same carbohydrate niche appears to be a promising strategy for colonization exclusion of *Salmonella*.

*Salmonella* also thrives in aerobic conditions, preferring to colonize when there are higher oxygen levels, such as in young chicks prior to the establishment of a stable microbiome (Litvak et al., 2019). This suggests that oxygen could be a limiting nutrient to consider in strategies aimed at enhancing colonization resistance. This desire for oxygen may drive inflammation observed in the early stages of *Salmonella* infection in chickens, where oxygen is released as a part of the host’s inflammatory response (Wigley, 2024).

*Salmonella* commonly inhabits the ceca of chickens, likely due to the nutrient rich environment and balanced pH found (Fanelli et al., 1971). It mainly resides in the cecal lumen of poultry but is also capable of colonizing the mucosal surfaces (Ijaz et al., 2021). Additionally, *Salmonella* can colonize tissues outside the gastrointestinal tract through its *Salmonella* pathogenicity islands (SPI-1 and SPI-2), which are groups of genes found within the chromosome that encode for multiple virulence factors (Shaji et al., 2023). These islands both provide a Type III secretion system (T3SS) as well as multiple invasins and adhesins, with SPI-1 enabling *Salmonella* to invade enterocytes and SPI-2 allowing intracellular survival within vacuoles of macrophages. The SPI-1 T3SS injectisome is composed of assembled proteins from SPI-1where the base is comprised of SpaP/Q/R/S and InvA; an outer ring made of InvG, PrgH, and PrgK; and needle made of PrgJ capped with SipD which regulates the secretion of proteins into the host cell with transmembrane proteins SipB and SipC acting as a translocon (Worley, 2025; Lou et al., 2019). Effector proteins are then secreted into the host cell AvraA, NF-κB inhibitor, SipA, actin-binding protein for membrane ruffling, SptP, actin reversal and downregulator membrane ruffling, and Sops, variety of functions such as cytoskeletal rearrangement, fluid secretion and formation of the *Salmonella* Containing vacuole (SCV). The SPI-2 T3SS injectisome is quite similar in design to the SPI-1 T3SS, however its effectors are different and specialized for increasing intracellular survival. The core subset of effectors shared across serovars are SseF, SseG, PipB, SteA, SifA, SteD, and PipB2 performing a wide array of functions from tethering the SCV to the Golgi network to recruiting host proteins to the SCV (Jennings et al., 2017). The SPIs allow *Salmonella* to travel through the hosts bloodstream and reach other favorable sites for colonization, such as the liver, spleen, and reproductive organs, providing special niches that enhance its ability to persist within the host (Matulova et al., 2013; Higginson et al., 2016; Buchmeier and Heffron, 1991). This spread to additional host tissues adds to the difficulty of controlling *Salmonella* in chicken products as contamination from feces is not the only source *Salmonella*. Spread to the reproductive organs also leads to the vertical transmission of *Salmonella* from hen to egg leading to retention of *Salmonella* in breeding stocks (Isah et al., 2024).

## Current preharvest *Salmonella* reduction efforts in poultry using probiotics

### Overview of preharvest interventions

Preharvest interventions for controlling *Salmonella* in poultry have become an integral part of the production process and are the first line of defense for consumers from foodborne pathogens (Logue et al., 2024). Current strategies for preharvest intervention include biosecurity, vaccination, and feed additives (Table 1). Establishing biosecurity is the most essential of all preharvest interventions as preventing the introduction of pathogens to a community is the best protection against it, however it does little if *Salmonella* is already present within the flock. Vaccination is also a common practice for poultry producers capable of reducing *Salmonella* prevalence within the flock in addition to reducing the amount shed by infected individuals, but efficacy can vary based on the serovars vaccinated against and route administration (Desin et al., 2013; Pan et al., 2024). Route of administration can also increase the cost of vaccination, with injection being laborious, while an oral live-attenuated vaccine can be easily administered but have the risk of reverting to a virulent form or lose efficacy over time (Bull, 2015; McWhorter and Chousalkar, 2018; Klose et al., 2023). Feed additives such as probiotics and competitive exclusion products are a preharvest control that many producers have begun to use to increase productivity and performance of their flocks. Furthermore, probiotic use may be a potential way of reducing the ability of *Salmonella* to colonize through competitive exclusion (Lutful Kabir, 2009; Abd El-Hack et al., 2022; Naeem and Bourassa, 2025).

**Table 1:** Preharvest interventions used in controlling bacterial pathogens for poultry production.

Preharvest intervention	Description	Examples	Reference
Biosecurity	A combination of management practices and protocols to prevent the transmission of pathogens to the flock through restricting movement of potential carriers.	Isolation of new animals Management of facility visitors Litter treatments Cleaning and disinfection	Logue et al. (2024), Souillard et al. (2024), and Bakin et al. (2025)
Vaccination/immunization - Inactivated vaccine - Live attenuated vaccine - Subunit vaccine	Whole bacterial cells made non-infectious by inactivation combined with an adjuvant Live bacteria altered to reduce virulence and mimic natural infection Specific immunogenic portion of bacteria administered	Nobilis®Salenvac ETC Poulvac®SE AviPro®Megan®Vac 1 Poulvac®ST Vaxxon®SRP®SE	Neelawala et al. (2024), Prakasan et al. (2026), Neelawala et al. (2024), Prakasan et al. (2026), Neelawala et al. (2024), and Prakasan et al. (2026)
Feed additives - Competitive exclusion products - Probiotics - Prebiotics - Synbiotics - Postbiotics - Organic acids/Fatty acids - Essential Oils	Fermented intestinal contents meant to alter microbiome to healthy adult state Live microorganisms that provide health benefits to their host Nondigestable dietary components that support beneficial bacteria Combination of probiotics and prebiotics mixed into a single administrable product Killed microbial cells or their metabolites used to obtain the benefits of probiotics without addition of microbes Weak acids possessing antimicrobial or immune modulatory properties Aromatic compounds from plants that possess antimicrobial properties	Broilact® Aviguard® *Lactobacillus* species *Bifidobacterium* species *Bacillus* species *Enterococcus* species Inulin Fructo-oligosacharides Resistant starches PoultryStar® Safmannan® Butyric acid Acetic acid Lactic acid Thymol Menthol Carvacrol	Naeem and Bourassa (2024), Naeem and Bourassa (2024), Ricke (2018), Naeem and Bourassa (2024), Naeem and Bourassa (2024), Gerzilov and Hristakieva (2025), and Naeem and Bourassa (2024)

### Competitive exclusion

The concept of competitive exclusion has been extensively researched in relation to *Salmonella* and poultry (Table 2). This practice is often attributed to Nurmi and Rantala, who demonstrated that administering cecal contents or anaerobically cultured alimentary contents from mature birds could effectively block *Salmonella* colonization in the guts of chicks (Pande et al., 2016; Nurmi and Rantala, 1973; Rantala and Nurmi, 1973). The mechanisms behind competitive exclusion can be summarized as niche-occupying effects, which include nutrient competition, production of bacteriocins and local environment alterations, inhibition of pathogen adhesion, and modulation of the host immune system (Figure 1; Hibbing et al., 2010). By administering organisms that occupy a pathogen’s targeted niches, these pathogens lose their ability to colonize the host.

**Table 2:** Research into competitive exclusion and *Salmonella* in poultry over 50 years.

Source	Purpose	Findings
Rantala and Nurmi (1973)	Prevent colonization of *Salmonella* Infantis in freshly hatched chicks through administration of various communities from different hosts	Administration of adult chicken alimentary contents both cultured and uncultured prevented colonization of *Salmonella* Infantis
Kolososki et al. (2024)	Examine difference in administration of aerobic vs. anaerobic culture of feces for competitive exclusion *S.* Heidelberg	Anaerobic culture of feces produced lower recovery of *S.* Heidelberg vs. aerobic and untreated birds
Kim et al. (2022)	Reduce *Salmonella* colonization through administration of a mixture of Lactic Acid Bacteria (LAB)	*Salmonella* was not found after administration of LAB mixture even when initially detected
Bailey et al. (1998)	Assess the effects of early exposure to *Salmonella* prior to administration of competitive exclusion product in hatcher using a seeder model	Early exposure of *Salmonella* prevented the reduction effect of competitive exclusion with only one seeder necessary for this effect
Ferreira et al. (2003)	Assessing the effectiveness of three different commercial competitive exclusion products at excluding *Salmonella* Kedougou from chicks	Mucosal starter culture and Aviguard were both effective at reducing *Salmonella* Kedougou colonization.
Palmu and Camelin (1997)	Determine efficacy of competitive exclusion product Broilact® at reducing *Salmonella* both on farm and in the processing pant	Broilact® did significantly reduce the number of positive flocks in both farm and processing plant
Corrier et al. (1998)	Examine the effect of simultaneous and delayed administration of competitive exclusion product with *Salmonella*	Simultaneous administration reduced number of *Salmonella* positive chicks. Delayed administration had less of a reduction.

**Figure 1:**
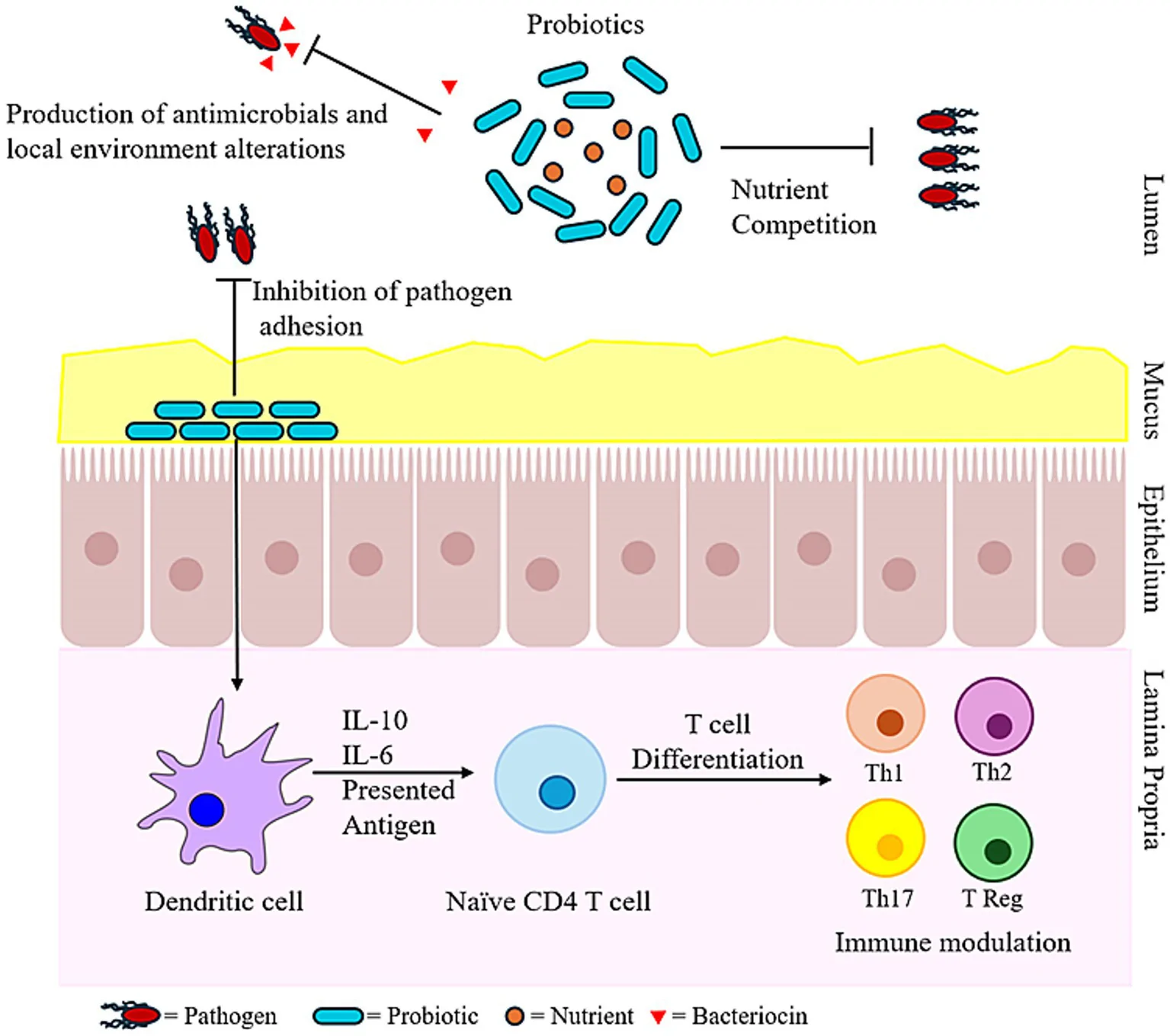
Mechanisms of probiotic competitive exclusion against pathogens. Probiotic organisms can inhibit the ability of pathogens to colonize the intestines through a variety of methods. Probiotics can compete for the same nutrients as pathogens as a form of indirect competition. Direct competition may be accomplished by the production of antimicrobials and alteration of local environment through pH alteration or other secreted metabolites that are unfavorable for pathogens. Probiotics may also occupy the same binding sites or physically occlude pathogens, preventing host cell invasion. Additionally, probiotics may interact with the host immune system through dendritic cells sampling the gut environment causing a cascade of interleukins leading to differentiation of T cells helping to modulate the host immune system.

Probiotics, whether single or multi-strains, are frequently administered to livestock as an alternative to antibiotics for increasing feed conversion and for providing competitive exclusion against enteric pathogens. Probiotics, as defined by the Food and Agriculture Organization of the United Nation World Health Organization, are “live microorganisms which when administered in adequate amounts confer a benefit to the host” (FAO and WHO, 2001). They typically consist of lactic acid bacteria, with significant emphasis on the inclusion of the family Lactobacillaceae as these organisms are commonly found in high abundance in mature and healthy poultry guts (Cheng et al., 2025). Lactobacillaceae are often found to have an antagonistic effect on *Salmonella* and other common pathogens in poultry often attributed to the production of organic acids and antimicrobial peptides causing a reduction or inhibition of their growth through acidification and membrane disruption, respectively (Abdel-Daim et al., 2013; Tian et al., 2024; Hai et al., 2021; Prabhurajeshwar and Chandrakanth, 2017).

Research on competitive exclusion of *Salmonella* often relies on the use of a few probiotic species or undefined/partially defined competitive exclusion products, but recent research has been directed more toward understanding why competitive exclusion products work and how to improve defined synthetic consortia (Spragge et al., 2023; Maurer et al., 2024). While the administration of individual probiotic organisms can reduce the overall *Salmonella* load in chickens, it does not appear to fully block colonization as seen with undefined competitive exclusion products (Lee et al., 2023). The reason for this difference is not entirely understood, but it is speculated that key members may be missing, likely strict anaerobes that colonize later in the development of a chicken. This is supported by evidence showing that administration of mature cecal contents is sufficient to inhibit *Salmonella* colonization in young chicks (Schneitz et al., 2016; Gast and Beard, 1989).

### Undefined vs. defined bacterial communities

A bacterial community is considered undefined when its exact strain composition is not fully known (Ibrahim et al., 2021). Undefined communities are typically derived from various environmental sources such as soil, sludge, or feces. The oral administration of an undefined community of bacteria, either through direct introduction of poultry cecal contents or by anaerobically culturing these contents and then feeding them to recently hatched chicks, is highly effective at limiting or even preventing the colonization of *Salmonella* (Nurmi and Rantala, 1973; Rantala and Nurmi, 1973; Yu et al., 1999). This has established fermentation products derived from chicken intestinal contents, such as Aviguard® or Broilact®, as an effective method for reducing *Salmonella* levels (Table 3). However, using undefined mixtures of bacteria has its drawbacks. One significant concern is batch variability, which can lead to inconsistent efficacy across different product batches (Salanitro et al., 1974; Waters et al., 2006; Lee et al., 2023). Moreover, the use of undefined products carries the risk of introducing pathogenic organisms if not properly monitored, potentially jeopardizing the health of the birds, the safety of consumers, and product availability by governing agencies (Waters et al., 2006). Additionally, undefined mixtures hinder our understanding of the exact mechanisms that limit or prevent infection, making it challenging for researchers to ascertain what is occurring within the bird (Lee et al., 2023). Advances in whole-genome metagenomic sequencing have helped to mitigate this issue by allowing genetic examination of competitive exclusion products and assist in potentially obtaining whole-genome sequences from undefined competitive exclusion products and genomically assess uncultivatable organisms for their ability to compete against pathogens. These third-generation sequencing approaches also allow for more thorough quality control of competitive exclusion products, aiding in confirming replication of the master seed used in the creation of the undefined product and monitor population drift.

**Table 3:** Effect of commercial and experimental probiotic competitive exclusion products against *Salmonella* in poultry.

Product	Nature	Microbial Taxa/Origin	Effects	Age/Breed
Broilact®	Undefined	Fermented Cecal Contents	Strong protective effect against *Salmonella* colonization (Schneitz et al., 2016)	Day-of-hatched Ross 508 Broilers
Aviguard®	Undefined	Fermented Cecal Contents	Reduction or elimination of *Salmonella* over 14 days (Nakamura et al., 2002)	1-day-hatched White Leghorn Layers
FloraMax-B11®	Defined	*Lactobacillus bulgaricus*, *Lacticaseibacillus casei*, *Lactobacillus cellobiosus*, *Limosilactobacillus fermentum*, *Lactobacillus helveticus*	Unable to block or reduce *Salmonella* colonization on its own (Schneitz et al., 2016). Reduces *Salmonella* when paired with perinatal supplement (Biloni et al., 2013)	Day-of-hatched Ross 508 Broilers Day-of-hatch Broilers
KMK3	Defined	*Lactobacillus brevis*, *Ligilactobacillus salivarius*, *Lactobacillus salivarius*	Increases antibody response and improves intestinal histological scores against *Salmonella* exposure (Boranbayeva et al., 2025). No prevention or reduction data shown.	1-day-hatched Broilers
SynCom^Bac10^	Defined	*Limosilactobacillus reuteri*, *Bacillus velezensis*, *Enterococcus faecium*, *Megamonas funiformis*, *Romboutsia lituseburensis*, *Anaerostipes caccae*, *Bacteroides xylanisolvens*, *Bifidobacterium pullorum*, *Enterobacter* sp., *Akkermansia muciniphila*	Improvement to phenotypic health indices such as stabilizing body weight gain and reducing organ damage indicators. Increased serum IgA levels against *Salmonella* (Zhang M. et al., 2025). No prevention or reduction data shown.	Day-of-hatch Breed not specified
PrimaLac®	Defined	*Lacticaseibacillus casei*, *Lactobacillus acidophilus*, *Enterococcus faecium*, *Bifidobacterium bifidum*	Lowered *Salmonella* counts in lower intestinal tract (Grimes et al., 2008)	Day-of-hatch Large White poults

A bacterial community is considered defined when well-characterized strains have been assembled into a reproducible community (Koch et al., 2026). Defined bacterial communities allow for better control over administered organisms, addressing issues linked to an undefined approach such as reproducibility and batch variability. However, there is still concern that some important member or aspect of the undefined community, which contributes to its effectiveness, may be overlooked by simplifying and defining the system (Maciag et al., 2023). Researchers have created multi-strain probiotic cocktails, typically composed of various lactic acid bacteria, yet these efforts rarely replicate the effectiveness of undefined competitive exclusion products when used to inhibit *Salmonella* within chickens (Schneitz et al., 2016; Grimes et al., 2008). Addition of more strains to a defined community is correlated with increased reduction of target pathogens, but it is difficult to ascertain why this may be occurring besides simply increasing metabolic coverage and redundancy and still does not achieve complete colonization inhibition (Spragge et al., 2023). More research is needed into why defined communities are unable to replicate the effectiveness of undefined competitive exclusion products for pathogen inhibition.

## Creating microbial consortia for inhibition of *Salmonella*

### Goals for consortium construction

The most common approach for creating a pathogen inhibiting consortium involves selecting consortium members based on their abilities to directly inhibit that pathogen. This is why lactic acid bacteria are commonly considered for consortium membership due to their known ability to kill and inhibit *Salmonella* (Schneitz et al., 2016; Grimes et al., 2008; Biloni et al., 2013; Boranbayeva et al., 2025). An alternative goal is to accelerate the maturation of the gut and its immune system to reach the infection resistant phenotype observed in the adult poultry gut, almost as though mimicking the effects of the administration of undefined fermented products. SynCom^Bac10^ is an example of this where the consortium was specifically constructed to capture the phylogenetic diversity observed in adult chickens, however they did not determine if *Salmonella* was still present within the chickens (Zhang Y. et al., 2025). Both goals conflict in their construction methodologies where the former considers only known inhibitors of the pathogen, while the latter looks at a *Salmonella* resistant community as a whole to create a microcosm. These methodologies of constructing consortia are referred to as bottom-up and top-down, respectively.

### Bottom-up versus top-down consortium construction

A bottom-up consortium design is the most common approach for consortium construction, especially for biomanufacturing, as it involves utilizing well characterized organisms as a base to work up from to achieve the functional goal of the community. Bottom-up focuses on using well-characterized strains and building the consortium up from those strains to perform a specific function such as biomass valorization or commercial manufacturing (San León and Nogales, 2022; Lyu et al., 2024). In the case of engineering a consortium for the inhibition of *Salmonella*, this would consist of organisms with known anti-*Salmonella* functions or known metabolisms that would compete for niche space with *Salmonella* (Figure 2A). Using well characterized organisms also allows for functional comparisons between consortium members mapping out metabolic pathways to promote specific anti-*Salmonella* functionality such as coverage of *Salmonella* consumed metabolites, lactic acid secretion, and bacteriocin production. In terms of using the bottom-up approach for competitive exclusion of pathogens, organisms that compete against the known consumed metabolites of the pathogen should be considered and can be carefully selected to provide the most overlap with *Salmonella* metabolism with the fewest organisms. Potential additions to the consortium may also be analyzed for their ability to function together and not compete with one another allowing for tightly defined control of what is occurring metabolically. Alternatively, focus could be placed on the support of known pathogen inhibitory elements produced by the consortium such as lactic acid or other bacteriocins or even to encourage the growth of specific non-culturable microbes such as segmented filamentous bacteria (Kim et al., 2022). This methodological design is not able to predict intra-consortium competition or interaction with the pathogen as not all bacterial genes are annotated or factor in metabolism alterations in the presence of other organisms, leading to differences in prediction versus tested application (Sanchez-Gorostiaga et al., 2019). This can often be seen as loss of certain members in the consortium over time in the host or reduced efficacy of the consortium compared to what may have been predicted due to deviations caused by higher order interactions of the present organisms. While the bottom-up approach is more rational in design and may lend itself well to microbial engineering, the ease of use and novel discovery chance of the less specific top-down approach makes it much more accessible and frequently used.

**Figure 2:**
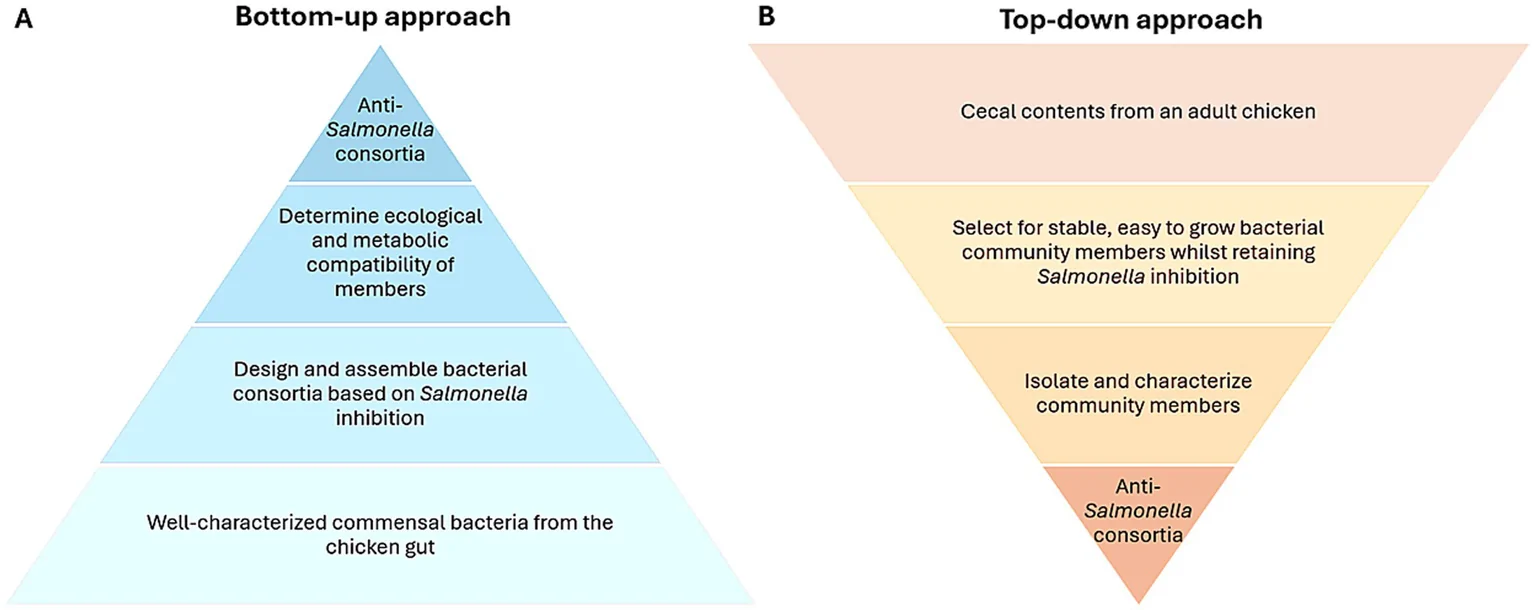
Comparison between **(A)** top-down and **(B)** bottom-up consortium construction methodologies for inhibition of *Salmonella*.

A top-down consortium lacks the rational design choices of the bottom-up approach’s choice designing a consortium for a specific purpose and instead attempts to replicate what would be found from the natural system it is derived from (San León and Nogales, 2022; Lyu et al., 2024). Using this approach for biomanufacturing often starts with identifying an existing natural microbiome that contains the desired function and optimizing it. In the case of *Salmonella* infection prevention in chickens, starting with known inhibitory communities such as cecal contents from mature birds, then working to identify which members appear to correlate most with inhibition may be the simplest approach (Figure 2B). The key benefit of the top-down approach is that no prior knowledge of community structure or dynamics are needed to start *in vitro* cultivation with only simple knowledge of the derived environment such as knowing the temperature, pH, dissolved oxygen concentration, and other similar factors are recommended. The simplistic nature of the top-down approach also reveals its flaws. Working with many unknowns leaves gaps in knowledge on how these organisms are functioning together, making dependency difficult to predict. Separating out the function of members may also be difficult due to the natural complexity of starting with unknowns.

### The middle-out approach for creating a *Salmonella* inhibiting consortia

A more comprehensive approach to consortium construction will likely involve a combination of the top-down and bottom-up approaches to generate a more effective consortium. This approach is known as middle-out allowing for both originally discussed approaches to make up for each other’s weaknesses (San León and Nogales, 2022; Lyu et al., 2024). This approach has proven its efficacy in the creation of synthetic microbial consortia for ammonia assimilation from wastewater treatment where a known *Psychrobacter aquimaris* was allowed to self-assemble a microbiome from active sludge to form a stable community (Zhang et al., 2021). Assembling a consortium in a similar way, but for a different function should also prove possible. There is a wealth of known inhibitors to *Salmonella* that may be used to generate a self-assembled community such as the Lactobacilli described previously that could be mixed with cecal contents from *Salmonella* resistant birds to enrich for members that will survive the interaction and be assessed for *Salmonella* inhibition. Alternatively, a more novel approach of taking known contributors of a typical healthy birds microbiome could be mixed with known *Salmonella* inhibitors and assessed for their ability to function together in a community and determine their ability to inhibit *Salmonella*. Organisms from the avian microbiome may be chosen based on their niche occupying properties in comparison to *Salmonella* where favor is placed on factors such as spatial occupation within the gut and metabolic profile for greater overlap and increased likelihood of competition with *Salmonella* supplemented with the additional backing of tested and known inhibitors to *Salmonella*.

## Screening and assessment of pathogen excluding consortia

### *In silico* modeling of *Salmonella* competitive exclusion

Computational modeling of bacterial interactions is a fast-growing field with great potential for the *in silico* prediction of metabolic exclusion of pathogens. One significant advancement in this area is the ability to quickly create genome-scale metabolic models (GEMs). A GEM encompasses an organism’s entire metabolism capacity, including all genes, gene-protein-reaction associations, metabolites, and transport reactions (Antonakoudis et al., 2020). With the increasing accessibility and accuracy of whole genome sequencing for bacteria through advances in third-generation sequencing approaches such as Oxford Nanopore or PacBio, obtaining complete bacterial sequences has become easier than ever, enabling the mass creation of GEMs (Figure 3). After genome assembly, the whole-genome sequences can be annotated into a protein sequence using protein annotation software, such as Prodigal, providing the protein framework for GEM creation (Hyatt et al., 2010). Subsequent computational programs, like CarveMe (Machado et al., 2018), can utilize this protein data to form a GEM.

**Figure 3:**
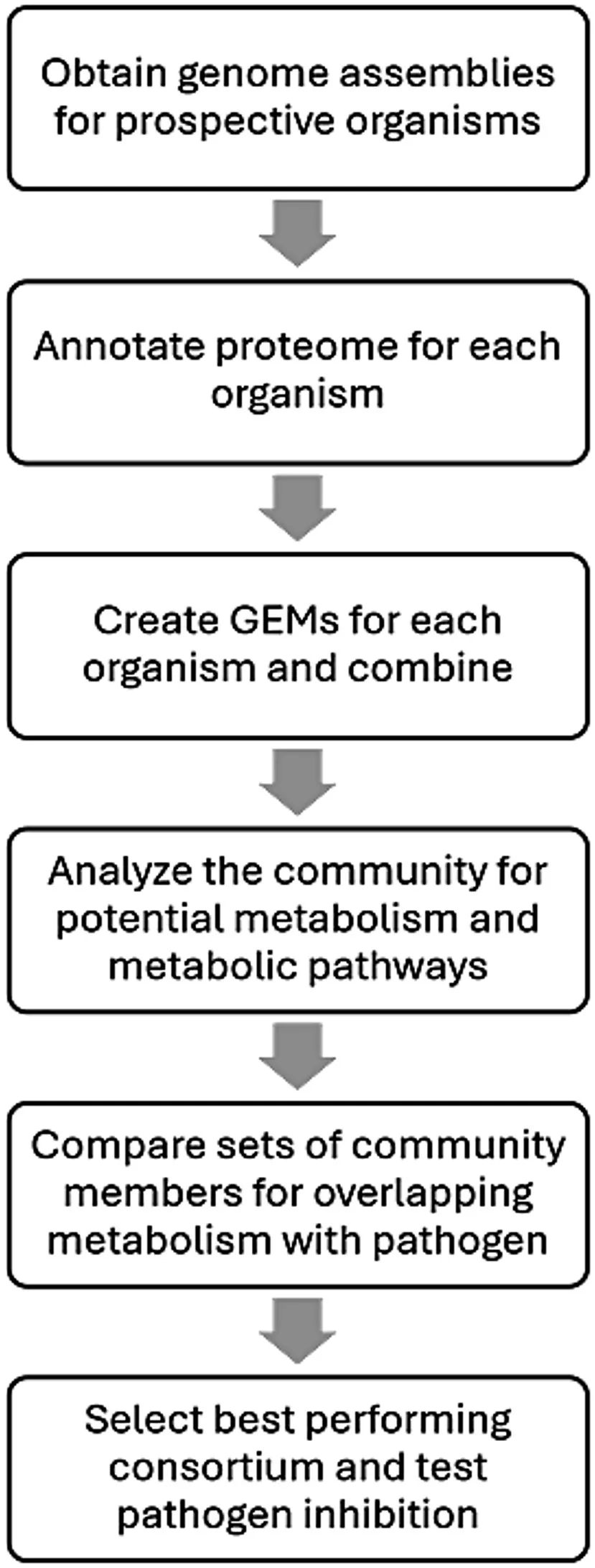
Proposed process for *in silico* selection of consortium members through metabolic competition.

Once GEMs are created, they can be combined and analyzed using various COBRApy (Ebrahim et al., 2013) based tools, such as CobraMod (Camborda et al., 2022), which provide flux balance analyses. These analyses can illustrate how metabolites are utilized throughout the metabolic network and allow for the visualization of metabolic pathways. Following the proposed reduction methodology of competitive exclusion, predictions can be made about which combinations of organisms are most effective in competition metabolically against specific pathogens based solely on the collected genomic information. This approach to consortium selection resembles a bottom-up methodology, where researchers sift through a pool of isolates derived from the chicken gut to identify organisms with metabolisms that directly compete or interfere with *Salmonella*. Additionally, known *Salmonella* inhibitors can be included in the consortium, with their GEMs incorporated into the flux balance analysis, leading to a more integrated consortium that may prove more effective.

With the rapid advancements occurring in Artificial Intelligence (AI) in over the last few years, AI-based consortium design is becoming a more prominent aspect of the field. The level of complexity involved in synthesizing all potential factors that may influence pathogen colonization is substantial, encompassing the host system, pathogen specifics, diet, housing conditions, the list of selected inhibitory organisms, and so on (Pickard et al., 2017). While machine learning (ML) can be used to generate predictions based on specific models, AI-based tools may allow for better synthesis of key factors for competitive exclusion generated from large quantities of ML data (Garcés-Ruiz et al., 2026). AI is likely to play a role in the future development of bacterial consortia and a few studies have already begun testing its ability to predict consortia for various purposes, but not for competitive exclusion (Liu et al., 2026; Alanazi et al., 2026). I*n vitro assessment of pathogen-excluding consortia.*

Once a consortium is established, it is beneficial to evaluate its efficacy and the ability of each member to work well together *in vitro* before moving on to *in vivo* testing. The efficacy of the consortium can be assessed in several ways: co-culture, exposure to cell free suspension (CFS) collected from the consortium, and colonization resistance (Van der Wielen et al., 2002; Abdel-Daim et al., 2013). Co-culturing typically involves inoculating and growing both the consortium and pathogen of interest in the same media simultaneously. This setup allows for the observation of direct competition between the consortium and the pathogen, in contrast to a monoculture of the pathogen. Pathogen colony forming units (CFUs) in the mixed culture can be measured using various methods, with the most common being the introduction of a plasmid into the pathogen. This plasmid may carry either a specific antibiotic resistance cassette that is absent in the consortium members or a fluorescent protein for detection by plate reader (Maurer et al., 2024). Colonization resistance involves initially inoculating the media with the consortium. After a set period, or once equilibrium is established, the pathogen is introduced into the same media to assess its ability to invade the system (Van der Wielen et al., 2002). The quantity of the pathogen is measured using the same methods applied in co-culturing. If the observed quantity of the pathogen falls below detectable levels, enrichment techniques can be employed to confirm its presence or absence. For *Salmonella*, various enrichment broths such as Rappaport-Vassiliadis *Salmonella* broth and Gram-negative broth, can be used to enhance detection of *Salmonella*. This enriched sample can then be plated on selective and identifying media such as XLT4 agar (Fagerberg and Avens, 1976; Miller et al., 1992).

Interactions among members of a consortium are essential for their viability and should be evaluated regardless of the specific pathogen of interest. The same principles used to test the effectiveness of the consortium in inhibiting pathogens can be applied here, but it is important to consider the outcomes of each individual member of the consortium and assess their viability. To compare each member individually, techniques such as disc diffusion, well diffusion, or agar plug diffusion can be employed to evaluate the formation of zones of inhibition, with larger zones indicating stronger antagonism among members (Balouiri et al., 2016). For a comprehensive comparison of all members simultaneously, they can be grown together, and their viability can be assessed using various methods. The simplest method would involve using specialized media for each member to obtain cell counts. Alternatively, relative abundance can be determined through 16S rRNA gene amplicon sequencing, while absolute quantification can be achieved using qPCR probes designed for each member to measure the copy number of genomic DNA present of each organism (Virgo et al., 2025; Chiarini et al., 2025; Lameira et al., 2024; Brugiroux et al., 2016). These relatively straightforward assays can be conducted in various settings, whether in a static tube, a chemostat, or cell culture. The only limitation is the degree to which one aims to mimic the host system. However, increasing the similarity to the host system reduces throughput and raises costs associated with equipment, materials, and time.

### *In vivo* testing and assessment of pathogen-excluding consortia

Once a consortium is sufficiently tested *in vitro* and proven to be stable, the next step is to shift to *in vivo* studies to demonstrate its efficacy. It is important to note that the breed of chicken can significantly influence how well the consortium colonizes or functions. Broilers and layers, for instance, are known to have different microbiomes, which can result from both dietary and genetic differences (Willson et al., 2022). For testing colonization resistance, chicks are typically given the consortium on the day-of-hatch and then inoculated with *Salmonella* after a delay of 24 to 48 h. This waiting period allows for the establishment of the consortium (Schneitz et al., 2016; Grimes et al., 2008; Zhang Y. et al., 2025). It is also essential to consider testing the administration of the consortium using commercial methods, such as a feed or drinking water additive, as this may alter the administered community by affecting its member’s viability and stress tolerance (Halder et al., 2024).

Many of the same techniques used in the *in vitro* testing may be employed for determining the presence and quantity of *Salmonella* within tested birds. It is advisable to sample multiple sites for *Salmonella* presence, as some serovars are known to colonize multiple tissue, including the cecum, liver, and spleen as well as less commonly affected tissues like bone marrow and muscle (Wang et al., 2024; Rimet et al., 2019). Enrichment is particularly important in this context, as *Salmonella* counts may fall below quantifiable levels using standard detection methods, especially if the administered bacterial consortium is performing well.

## Conclusion

Bacterial consortia provide a unique avenue for the inhibition of foodborne pathogens of concern in chickens. *Salmonella* poses a particular challenge for elimination in poultry due to its seemingly commensal nature in older birds. Standard treatment practices like vaccination are not completely effective at eliminating *Salmonella* creating a need for alternative approaches. Probiotics and eventually designed bacterial consortiums are a promising field for the control of *Salmonella* in poultry given the efficacy of undefined cecal contents in the inhibition of *Salmonella* colonization in young birds. While undefined cecal content derived communities of bacteria work well in preventing the colonization of *Salmonella*, providing an alternative in the form of a defined community will help to reduce potential batch variation and allow for quicker approval by governing agencies for use in agriculture. *Salmonella* has the potential to be eliminated using a well-defined consortium of bacteria derived from the *Salmonella* resistant adult chicken gastrointestinal tract following the principles of competitive exclusion. Recent advances in computation and the ease of whole-genome sequencing of bacterial isolates allow for faster screening of potential candidates for *Salmonella* niche occupation and inhibition through comparison of those organisms GEMs. Sequencing advances also allow for better characterization of undefined competitive exclusion products potentially aiding in extracting important uncultivatable organisms for further analysis and eventual cultivation. Biomanufacturing research has also helped to inform consortium construction methodologies such as using a middle-out approach, making consortium construction a promising future for the probiotics research in poultry.
